# Uniaxial Compressive Failure Characteristics and Constitutive Modeling of Fractured Coal Mass Under Different Strain Rates

**DOI:** 10.3390/ma18092000

**Published:** 2025-04-28

**Authors:** Zihao Feng, Haitao Li, Xiaoshan Shi, Chunyuan Li, Honghui Yuan, Zhengyi Li, Huaguang Liu

**Affiliations:** Deep Mining and Rock Burst Research Institute, Chinese Institute of Coal Science, Beijing 100013, China; xiaofeng0686@163.com (Z.F.);

**Keywords:** CT scan, uniaxial compression, dynamic characteristics, patterns of destruction, constitutive model

## Abstract

To investigate the effects of different strain rates and fracture densities on the mechanical behavior of coal, CT scanning was employed to quantify fracture content in coal specimens. Uniaxial compression tests were conducted to analyze the mechanical characteristics of coal, followed by the establishment of a statistical damage constitutive model for fractured coal. The results demonstrate: (1) The compressive strength of coal specimens shows a positive correlation with increasing strain rate, while the elastic modulus exhibits an initial decrease followed by an upward trend. Compressive strength displays a negative correlation with fracture density. Under a strain rate of 0.001 s^−1^, the elastic modulus decreases significantly with increasing fracture density, whereas this trend becomes less pronounced at higher strain rates. Notably, compressive strength demonstrates greater sensitivity to fracture density variations. (2) Within the dynamic strain rate range of 0.001 s^−1^~0.05 s^−1^, the fractal dimension of fragmented coal particles ranges from 1.0 to 1.3. Both the average mass of ejected fragments and the mean fractal dimension of fractured particles increase progressively with strain rate elevation, indicating enhanced non-uniformity in particle size distribution. (3) Significant correlations exist between Weibull distribution parameters (*F*_0_, *m*) and strain rate/fracture density. A critical threshold emerges at 0.01 s^−1^ strain rate, where *F*_0_ and *m* exhibit opposite variation trends before and after this threshold. Similarly, a fracture density threshold between 0.3%~0.45% is identified, with *F*_0_ and *m* demonstrating contrasting evolution patterns below 0.3% and above 0.45% fracture density under increasing strain rates. (4) Based on the established relationships between Weibull parameters (*F*_0_, *m*), strain rate, and fracture density, the dynamic statistical damage constitutive model for coal was modified. A systematic methodology for determining parameters in the revised model was subsequently proposed.

## 1. Introduction

With the continuous increase in coal resource mining depths, the safety and efficiency of mining engineering face severe challenges [1,2,3]. The crux lies in the fact that mining activities disrupt the original in situ stress equilibrium, triggering a complex adjustment process involving stress redistribution and energy transfer. During this process, the dynamic mechanical properties and deformation-failure characteristics of coal-rock masses not only exhibit significant strain rate effects, but their inherent structural defects such as pre-existing microcrack networks also critically influence their mechanical responses [4,5,6]. The coupling of these two characteristics not only leads to nonlinear, multi-stage complex failure behaviors in coal-rock masses, but also serves as a crucial factor inducing dynamic disasters such as rockbursts. Therefore, establishing a multi-factor coupled damage constitutive model for coal-rock and revealing the synergistic mechanism of strain rate-fracture density dual factors could provide critical theoretical support for safe and efficient deep coal mining.

Current research methods for investigating the mechanical properties and failure characteristics of fractured coal-rock under dynamic loading primarily include experimental studies and numerical simulations [7,8,9]. These approaches have revealed significant correlations between coal-rock strength, deformation parameters, and strain rate/fracture characteristics. Wen Xiaozhe et al. [10] conducted Brazilian splitting tests on sandstone under medium-low strain rate disturbance loads, verifying the existence of disturbance load stress thresholds and finding that disturbance-induced damage degree positively correlates with pre-static load levels. Li Haitao [11] designed multi-strain rate mechanical experiments, revealing that the unique mechanical behaviors of coal under rate effects result from the combined action of crack development and bearing structure fracture. Tu Min et al. [12] performed true triaxial single-free-face tests on coal-rock under different loading rates, elucidating the influence mechanism of loading rate on dynamic failure. Li Chunyuan et al. [13] conducted triaxial loading-unloading-seepage tests, discovering that post-unloading crack evolution closely relates to fracture patterns. Huang Dongmei et al. [14] investigated damage evolution characteristics of rock samples with three types of crack defects through uniaxial compression numerical simulations. Yuan et al. [15] systematically investigated the influence of closed fissure dip angle, length, and spatial distribution on rock mechanical behavior, proposing a residual energy index to evaluate energy evolution characteristics. These studies demonstrate that the mechanical properties of fractured coal-rock vary significantly with different strain rates and fracture characteristics during loading damage processes.

Furthermore, research on dynamic constitutive relationships of coal-rock has attracted extensive attention. Zheng Yu et al. [16] established a dynamic strength statistical damage constitutive model for coal-rock based on the relationship between Weibull distribution parameters and strain rate. Zhang Liang et al. [17] developed a coal-rock damage-fracture model considering three-parameter Weibull distribution, gas effects, and effective stress through CT scan reconstruction and statistical strength theory. Zhang Chao et al. [18] proposed a static-dynamic statistical damage constitutive model incorporating rock damage thresholds, applicable for simulating rock deformation-failure processes under different strain rates. Wang Kai et al. [19] constructed an energy-damage constitutive model for coal-rock composite specimens under cyclic loading based on energy dissipation principles. Cao Wengui et al. [20] improved existing rock dynamic strength criteria to establish a nonlinear dynamic strength criterion reflecting strain rate effects, developing a rock dynamic statistical damage constitutive model with parameter determination methods based on dynamic triaxial test curves. Yan et al. [21] established a dynamic damage constitutive model incorporating strain-rate effects and micro–macro fissure coupled damage, comprehensively characterizing the coupled influence of strain rate and fissure damage on mechanical responses. He et al. [22] developed a numerical model by coupling cohesive zone elements with adjacent finite elements using identical continuum damage mechanics (CDM) formulations. Validation confirmed its capability to accurately represent elastic energy storage/release mechanisms and damage energy dissipation processes. Azadi et al. [23] compared the generalized Hoek–Brown criterion, strength degradation model (SDR), and CDM approaches, revealing that SDR and CDM significantly enhance simulation accuracy and applicability for weak rock masses through modeling nonlinear irreversible behavior and microscopic damage propagation. These methods provide improved long-term stability predictions in geotechnical engineering. Current research indicates that statistical damage theory remains the mainstream approach for modeling rock deformation-failure processes. The distinct stress–strain curves obtained under different strain rates or fracture densities necessitate further analysis of statistical damage simulation methods considering strain rate and fracture density variations, holding significant theoretical and engineering value.

Therefore, this study employs CT scan reconstruction and uniaxial compression tests to analyze the mechanical properties and fragmentation fractal characteristics of coal under different strain rates and fracture densities. By proposing a strength-type statistical damage constitutive model based on coal’s dynamic mechanical properties, this research aims to reveal the failure mechanisms of deep fractured coal under loading and provide scientific guidance for preventing dynamic disasters in deep mining, offering both theoretical and practical significance.

## 2. Experimental Equipment and Protocol

### 2.1. Specimens and Equipment

The coal specimens used in this study were collected from Xinjulong Coal Mine in Heze, China. Following the International Society for Rock Mechanics (ISRM) suggested methods, 16 cylindrical specimens (φ50 mm × 100 mm) were drilled and processed in the laboratory. The end surfaces of all specimens underwent precision grinding and polishing processes using a dedicated grinding apparatus developed in-house by the China Coal Research Institute, achieving surface parallelism within 0.03 mm and perpendicularity to the specimen axis, with a maximum deviation of ≤0.3°.

**Figure 1:**
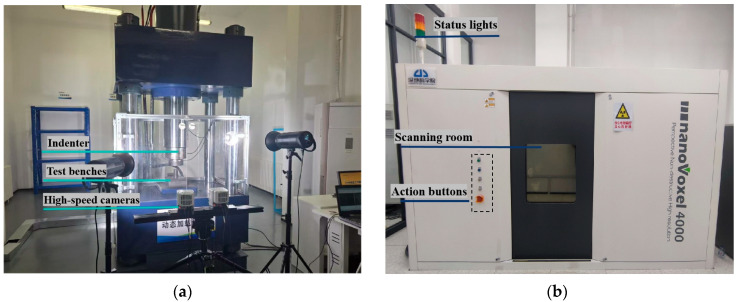
Test equipment: (**a**) Single-axis dynamic load mechanics test equipment; (**b**) Coal and rock CT scan and multi-field coupling system.

To analyze the dynamic mechanical properties, failure modes, and their relationships with internal structures under different loading rates, a uniaxial dynamic loading mechanical testing system and a coal-rock CT scanning and multi-field coupling system (Figure 1), both independently developed by the China Coal Research Institute, were utilized. The uniaxial dynamic loading system supports static loading tests, fatigue loading tests, and axial loading up to 2000 kN, with a maximum impact velocity of 25 mm/s and a minimum displacement loading rate of 0.01 mm/min. It features multiple waveform inputs, multi-data transmission channels, and static/dynamic dual-channel acquisition cards with a maximum sampling frequency of 10 kHz. The CT scanning and multi-field coupling system achieves a scanning resolution of 2 μm and provides a maximum axial pressure of 200 MPa, maximum confining pressure of 25 MPa, and displacement pressure of 35 MPa, enabling non-destructive detection of internal microstructures such as fractures and mineral distributions.

### 2.2. Experimental Protocol

**Figure 2:**
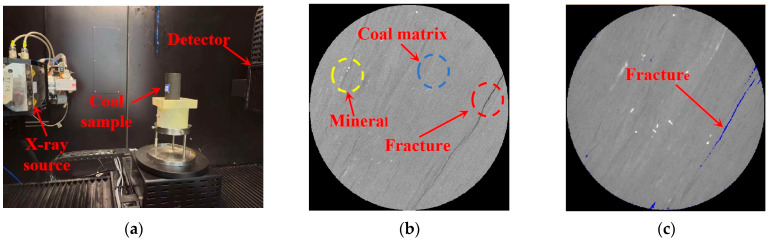
Fissure extraction process: (**a**) CT scan; (**b**) 2D slice; (**c**) threshold segmentation.

To analyze the natural fracture structure content within different coal specimens, pre-experimental procedures were conducted as follows: Prior to uniaxial compression testing, coal specimens were scanned using a multi-physics coupled CT system under laboratory conditions, as illustrated in Figure 2a. Scanning parameters were set at 200 kV voltage, 180 μA current, and 25.14 μm resolution. The acquired CT datasets underwent sequential processing: First, data reconstruction was performed using VoxelStudio Recon software (2022 version), followed by 3D specimen reconstruction in Avizo software (2019.1) to generate 2D grayscale slices (Figure 2b). The grayscale values ranged from 0 (absolute black) to 65,535 (pure white), with specific microstructural features identified through chromatic annotations: yellowish-white regions (yellow circles) representing mineral inclusions, blue-circled areas indicating coal matrix, and black linear structures within red circles corresponding to fractures. Fracture extraction was achieved through threshold segmentation, with grayscale range 0–24,300 empirically determined for fracture identification in batch-tested specimens (Figure 2c). Fracture density was quantified as the volumetric ratio of segmented fractures to total specimen volume.

Based on fracture density, the CT-scanned specimens were classified into four groups: ultra-low (C-1: 0–0.1%), low (C-2: 0.1–0.2%), medium (C-3: 0.3–0.45%), and high (C-4: 0.45–0.9%), with four specimens per group (e.g., C-1-1 to C-1-4 for Group C-1). Field investigations by geomechanics researchers have revealed that near-source dynamic loading strain rates during mining-induced seismicity typically range from 10^−3^ to 10^−1^ s^−1^. Consequently, loading conditions exceeding 10^−3^ s^−1^ strain rate can be classified as coal mine dynamic loading. Investigating the deformation and failure mechanisms of coal-rock masses within this strain rate regime holds critical implications for prevention and mitigation strategies against coal mine dynamic disasters [24]. In alignment with International Society for Rock Mechanics (ISRM) testing standards, which mandate a minimum triplicate specimens per test group for characterizing uniaxial compressive strength and compressive deformation behavior, this experimental framework ensures statistically validated results when studying coal-rock mechanical responses under varying strain rate conditions. A 4 × 4 cross-designed uniaxial compression test matrix was implemented, where specimens from each group were tested under four strain rates (0.001 s^−1^, 0.01 s^−1^, 0.03 s^−1^, and 0.05 s^−1^). The failure processes were recorded using a high-speed camera (Phantom® S991 machine vision high-speed camera from Vision Research in Wayne, NJ, USA).

To investigate the coupled effects of fracture density and strain rate on mechanical behavior, the uniaxial compressive strength (*σ_max_*) and elastic modulus (*E*) were analyzed. Table 1 summarizes the CT scanning results, uniaxial compression test results, and calculated parameters, where *η* denotes fracture density, *σ_max_* represents uniaxial compressive strength, and *E* indicates elastic modulus.

To ensure the reliability of fracture density classification, a hypothesis testing procedure was conducted on the fracture grouping scheme. The null hypothesis assumes no significant differences among four fracture density groups at a significance level of α = 0.05. Through one-way ANOVA analysis of fracture density parameters, the following statistical results were obtained (Table 2):

The calculated *p*-value of 4.1 × 10^−7^ substantially exceeds the critical threshold (*p* < 0.05), leading to rejection of the null hypothesis and confirming statistically significant inter-group differences. Furthermore, the exceptionally low intra-group variances (0.0007~0.0223) demonstrate superior homogeneity within each classification category, thereby validating the rationality of the proposed fracture density classification system.

## 3. Analysis of Dynamic Failure Process in Fractured Coal

### 3.1. Dynamic Stress–Strain Curves

**Figure 3:**
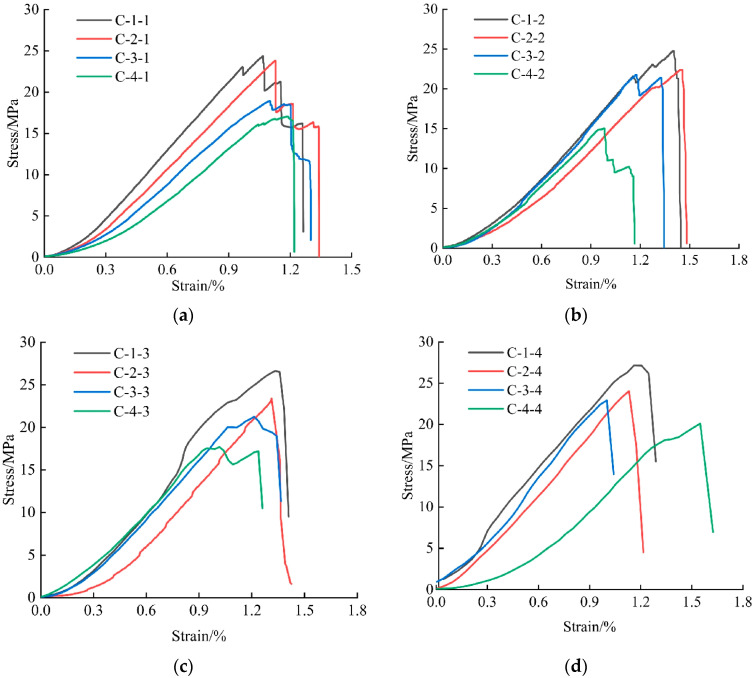
Stress–strain curve of coal sample: (**a**) 0.001 s^−1^; (**b**) 0.01 s^−1^; (**c**) 0.03 s^−1^; (**d**) 0.05 s^−1^.

Stress–strain curves under different loading rates were plotted based on experimental data (Figure 3). For specimens in low fracture density groups (C-1 and C-2) tested at 0.001 s^−1^ or strain rates of 0.01–0.05 s^−1^, the elastic deformation stage prolonged with increasing fracture density. However, this trend became less pronounced for high fracture density groups (C-3 and C-4) under 0.01–0.05 s^−1^. The results suggest that while the elastic deformation stage generally extends with fracture density, strain rate introduces complexity through coupled interactions with fractures.

### 3.2. Dynamic Mechanical Properties

**Figure 4:**
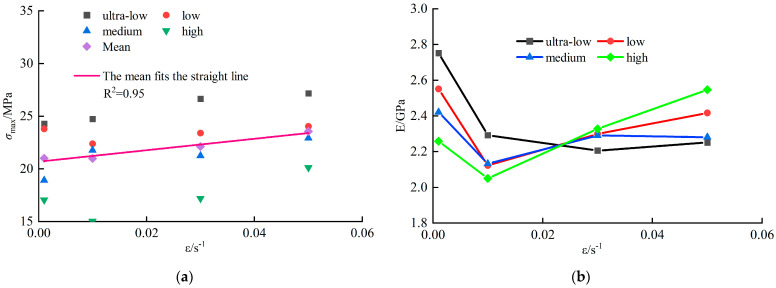
Mechanical properties of coal sample under different strain rates: (**a**) Compressive strength at different strain rates; (**b**) Modulus of elasticity at different strain rates.

As shown in Figure 4a, the uniaxial compressive strength (*σ_max_*) of coal specimens ranged from 15–28 MPa, exhibiting an overall upward trend with increasing strain rate. A linear fit (R^2^ = 0.95) confirms a strong positive correlation between *σ_max_* and strain rate.

Figure 4b reveals that the dynamic elastic modulus (*E*) of coal specimens varied between 1.9–2.8 GPa. For specimens across fracture density groups, E initially decreased and then increased with rising strain rate. Specifically, E decreased by an average of 11.9% as strain rate increased from 0.001 s^−1^ to 0.01 s^−1^, followed by a gradual increase from 0.01 s^−1^ to 0.05 s^−1^. Higher E values indicate greater deformation stiffness and enhanced resistance to failure.

**Figure 5:**
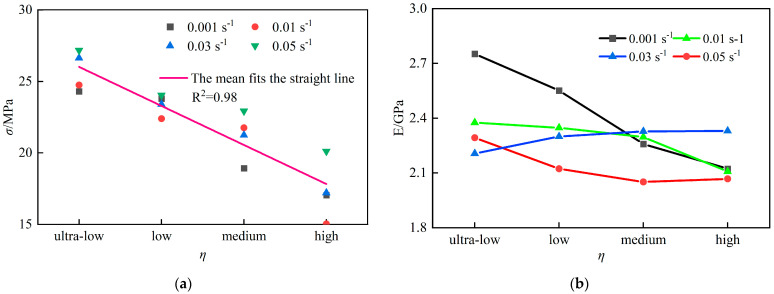
Mechanical properties of coal sample under different fracture rates: (**a**) Compressive strength at different fracture rates; (**b**) Modulus of elasticity at different fracture rates.

The relationship between *σ_max_* and fracture density (*η*) is illustrated in Figure 5a. The average *σ_max_* decreased from 25.70 MPa to 17.35 MPa (32.5% reduction) with increasing *η*, and a linear fit (R^2^ = 0.98) confirms a significant inverse correlation.

Figure 5b demonstrates the evolution of *E* with *η*. At 0.001 s^−1^, E decreased by 24.8% (from 2.75 GPa to 2.25 GPa) as *η* increased. In contrast, under 0.01–0.05 s^−1^, *E* declined only 6.6% (from 2.25 GPa to 2.10 GPa). This indicates that fracture density more critically impacts strength than stiffness, while strain rate amplifies the coupling effects between defects and mechanical responses.

### 3.3. Dynamic Failure Characteristics

**Figure 6:**
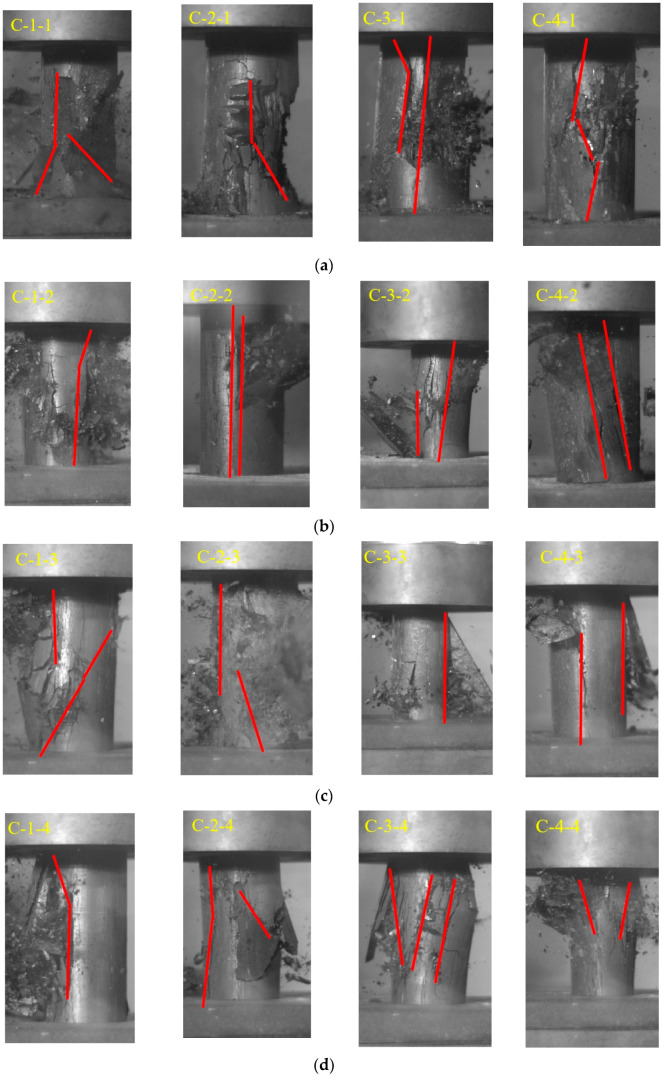
Failure mode of coal and rock: (**a**) 0.001 s^−1^; (**b**) 0.01 s^−1^; (**c**) 0.03 s^−1^; (**d**) 0.05 s^−1^.

During the loading phase of the tests, a Vic high-speed camera system was used to monitor the failure process of the coal samples in real time. Due to differences in the mechanical response characteristics of coal-rock under varying loading rates, typical failure features at different moments for each coal sample were selected for comparative and comprehensive analysis, as shown in Figure 6.

From Figure 6, it can be observed that at a strain rate of 0.001 s^−1^, the coal-rock samples primarily exhibited shear failure and composite failure. When the strain rate increased to 0.01 s^−1^, the coal samples still mainly failed by shear and composite failure, though sample C-2-2 displayed cleavage failure. At a strain rate of 0.03 s^−1^, samples C-1-3 and C-2-3 experienced composite failure, while samples C-3-3 and C-4-3 underwent cleavage failure. At a strain rate of 0.05 s^−1^, the coal samples predominantly exhibited shear failure and composite failure. These results indicate that within the strain rate range of 0.001 s^−1^ to 0.05 s^−1^, the effects of strain rate and fracture porosity on the failure mode of the coal samples are not significant; instead, the dominant failure mode is governed by the geometric characteristics of the internal fracture network and bedding planes [13,15].

**Figure 7:**
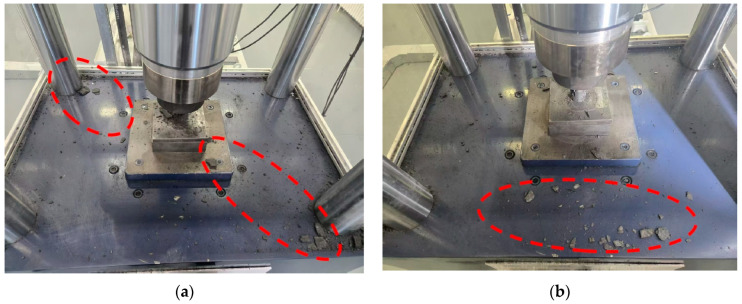

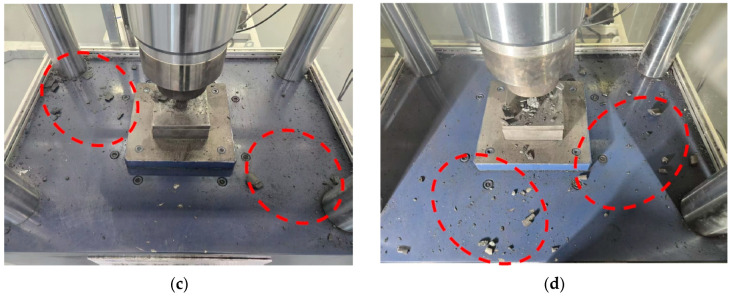
Schematic diagram of coal sample ejection characteristics under different loading rates: (**a**) 0.001 s^−1^; (**b**) 0.01 s^−1^; (**c**) 0.03 s^−1^; (**d**) 0.05 s^−1^.

Under different strain rate conditions, the ejection distance of the coal fragments is mainly concentrated within the red-circled area of the blue operating platform, and the ejection distance generally increases with strain rate. As a manifestation of dynamic failure, the ejected coal fragments show slight variations in particle size under different strain rate conditions, as illustrated in the red circle in Figure 7. To more intuitively analyze and compare the degree of fragmentation of the coal samples under different loading rates, the fractal dimension *D* is calculated based on the relationship between fragment mass and equivalent side length. This quantifies the distribution characteristics of the fragments [25]. A higher fractal dimension indicates a larger number of fragments with smaller volumes, signifying a higher degree of fragmentation. The calculation formula is as follows.

*D* = 3 − *b*, (1)

(2) $$b=\frac{\mathrm{lg}\left(M_{L_{eq}}/M\right)}{\mathrm{lg}\left(L_{eq}\right)},$$

In the equation, *M_Leq_* represents the cumulative mass of fragments with equivalent side lengths smaller than *L_eq_*; *M* denotes the fragment mass within the measurement range; *D* is the fractal dimension of the fragments; and *b* is the slope of the lg(*M_Leq_*/*M*) versus lg(*L_eq_*) curve on a double-logarithmic plot.

Due to the high integrity of the coal samples following localized failure on the loading platform, no clear trend was observed across the entire size range, consistent with previous studies [26]. To reduce the influence of outliers on the slope and correlation coefficient of the fitted lg(*M_Leq_/M*) and lg(*L_eq_*) curves, the size distribution of the coal sample fragments was characterized using standard sieves with apertures of 5, 16, 25, and 31.5 mm. The fragments were sieved and weighed, and the fractal dimensions of coal samples tested at the same strain rate were averaged. The results of the fragment sieving tests and the average fractal dimensions are presented in Table 3.

Table 2 indicates that at lower strain rates, the fragment mass is relatively uniform across the various size intervals. As the strain rate increases, the overall mass of the ejected coal fragments gradually rises. Notably, the fragment mass in the 25–31.5 mm and <5 mm ranges exhibits a significant increase, while the mass increase in the 5–25 mm interval is less pronounced, suggesting a more heterogeneous fragment size distribution. Moreover, the average fractal dimension of the fragments gradually increases with rising strain rate.

At lower strain rates, the coal undergoes effective self-adjustment, with a substantial portion of the accumulated elastic energy being utilized for crack propagation and performing work on localized fracture zones, which results in a higher degree of overall fragmentation. Under these conditions, the residual elastic energy is limited, leading to the ejection of only the deeply fragmented coal, hence the ejected mass is lower and the ejection distance is shorter. In contrast, at higher strain rates, the peak instability, fragmentation degree, ejected mass, and ejection kinetic energy of the coal are significantly enhanced. This is because a higher strain rate partially suppresses the full propagation of internal cracks, allowing the coal to sustain greater deformations while enhancing its overall load-bearing capacity, thereby accumulating higher elastic potential energy. Specifically, during the initial phase of instability, the shallow coal is typically ejected as larger blocks with a greater ejection distance; however, at the moment of instability, the increased work done on fragmentation results in a greater number of fine fragments, thereby increasing the overall ejected mass. Thus, the increase in strain rate exerts a positive stimulative effect on the dynamic failure of coal.

## 4. Dynamic Damage Constitutive Model for Fractured Coal

### 4.1. Constitutive Model Development

In rock mechanics, based on the assumption that rock consists of numerous micro-elements, representative volume elements (RVEs) are homogenized from heterogeneous micro-blocks. Statistical strength theory is then applied to describe the strength distribution of these micro-elements, tracking their progressive failure and cumulative damage to establish macroscopic deformation-failure evolution laws via continuum damage mechanics. The stochastic nature of internal fractures is typically characterized by a Weibull distribution with damage threshold effects.

As a heterogeneous material containing randomly distributed fractures, coal is idealized as an assembly of micro-elements that encapsulate defects at a mesoscale while behaving as isotropic continua at the macroscale. Key assumptions include: (1) Coal exhibits approximate isotropy even under damage. (2) Micro-elements obey Hooke’s law until failure. (3) Micro-element strengths follow a Weibull distribution, defined by the probability density function:

(3) $$P\left(F\right)=\frac{m}{F_{0}}{\left(\frac{F}{F_{0}}\right)}^{m-1}\exp\left[-{\left(\frac{F}{F_{0}}\right)}^{m}\right],$$

where *F* is the micro-element strength variable, *F*_0_ and *m* are Weibull parameters reflecting material properties.

Coulomb (M–C) criterion demonstrates better applicability to experimental data in engineering practice compared to the Lade–Duncan (L–D) criterion, the M–C criterion also slightly outperforms the Drucker–Prager (D–P) criterion under varying confining pressures [17]. However, the D–P criterion is widely adopted for characterizing micro-element strength due to its robust performance. Thus, this study employs the D–P criterion [27] to define *F* as follows:

(4) $$F=\alpha I_{1}+\sqrt{J_{2}},$$

(5) $$\left\{\begin{array}{l} \alpha =\frac{\sin\varphi }{\sqrt{9+3\sin^{2}\varphi }}\\ I_{1}=\frac{\left(\sigma_{1}+2\sigma_{3}\right)E\varepsilon_{1}}{\sigma_{1}-2\mu \sigma_{3}}\\ \sqrt{J_{2}}=\frac{\left(\sigma_{1}-\sigma_{3}\right)E\varepsilon_{1}}{\sqrt{3}\left(\sigma_{1}-2\sigma_{3}\right)} \end{array}\right.,$$

where *α* is the micro-element strength parameter, *ϕ* is the internal friction angle of the rock, *I*_1_ is the first invariant of the stress tensor, and *J*_2_ is the second invariant of the deviatoric stress tensor.

Under uniaxial compression (*σ*_3_ = 0), substituting Equation (5) into Equation (4) yields the simplified micro-element strength expression:

(6) $$F=\left(\alpha +\frac{1}{\sqrt{3}}\right)E\varepsilon_{1},$$

Based on continuum damage mechanics and the concept of representative volume elements (RVEs), the damage variable *D* is defined as the ratio of damaged micro-elements (*N_f_*) to the total number of micro-elements (*N*):

(7) $$D=N_{f}/N,$$

(8) $$N_{f}=\int_{0}^{F}NP\left(x\right)dx,$$

Incorporating Equations (3) and (8) into Equation (7), the damage variable *D* is expressed as:

(9) $$D=1-\exp\left[-{\left(\frac{F}{F_{0}}\right)}^{m}\right],$$

Following Lemaitre’s strain equivalence hypothesis [23], the damage constitutive equation for conventional triaxial compression is derived:

(10) $$\sigma_{1}=E\left(1-D\right)\varepsilon_{1},$$

(11) $$E=\frac{\sigma_{1}}{\varepsilon_{1}},$$

(12) $$\mu =\frac{\varepsilon_{3}}{\varepsilon_{1}},$$

where *E* and *μ* are the elastic modulus and Poisson’s ratio, respectively, and *ε*_1_ and *ε*_3_ denote axial and radial strains.

When the load exceeds the strength threshold of micro-elements, localized damage initiates and propagates, leading to macroscopic yield and failure. By assuming that micro-element damage follows a probabilistic distribution governed by statistical strength theory, the damage variable *D* can be rigorously defined. The resulting damage model is expressed as:

(13) $$\sigma_{1}=E\left(1-D\right)\varepsilon_{1}=E\varepsilon_{1}\exp\left[-{\left(\frac{F}{F_{0}}\right)}^{m}\right],$$

### 4.2. Model Calibration

Parameters *F*_0_ and *m* are determined via the extremum method using peak stress (*σ_max_*) and strain (*ε_max_*) from uniaxial tests. By differentiating and simplifying the constitutive equations, *F*_0_ and *m* are derived as:

From the established constitutive model, the key to model construction lies in determining two unknown parameters: *F*_0_ and *m*. In uniaxial compression tests, peak stress and corresponding strain can be directly measured, enabling parameter calibration via the extremum method. By setting the derivatives of the multivariate constitutive equations to zero at peak conditions (Equations (6) and (13)), simplified expressions for *F*_0_ and *m* are derived.

(14) $$F_{0}=\left(\alpha +\frac{1}{\sqrt{3}}\right)E\varepsilon_{m}m^{\frac{1}{m}},$$

(15) $$m=-\frac{1}{\ln\left[\sigma_{\max}/E\varepsilon_{m}\right]},$$

The experimental program utilized coal specimens from a single geological batch, with measured internal friction angles (*φ*) distributed between 33–36°, yielding a mean value of 35°. Sensitivity analysis revealed <2% deviation in (UCS) predictions when *φ* was varied between 30–40°, demonstrating limited model sensitivity to this parameter. Consequently, the mean *φ* value of 35° was adopted for subsequent constitutive modeling to optimize computational efficiency while maintaining engineering accuracy. Parameters *F*_0_ and *m* were calculated via Equations (14) and (15), with results listed in Table 4.

**Figure 8:**
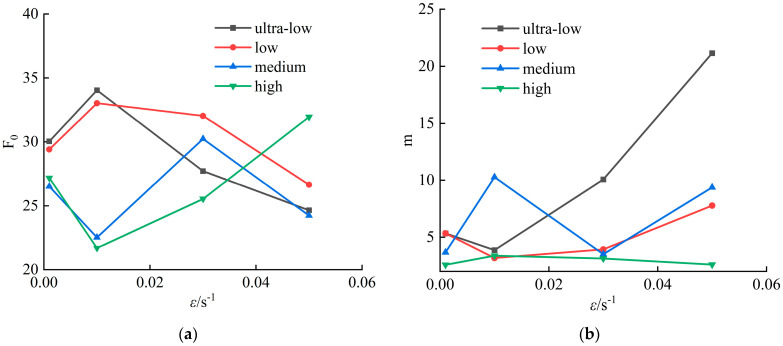
Parameters *F*_0_ and *m* vary with strain rate: (**a**) *F*_0_ varies with the strain rate; (**b**) *m* varies with the strain rate.

From the constitutive model calculation results in Table 3, it can be observed that the parameters *F*_0_ and *m* exhibit a certain correlation with the strain rate and fracture porosity. To illustrate this relationship more clearly, a line chart is plotted with the strain rate as the horizontal axis, as shown in Figure 8.

From Figure 8, it can be seen that for the C-1 and C-2 groups with low and extremely low fracture porosity, the parameter *F*_0_ increases first and then decreases with increasing strain rate; for the C-3 group with medium fracture porosity, *F*_0_ exhibits a wavy fluctuation; and for the high fracture porosity group, C-4, *F*_0_ decreases first and then increases. Similarly, the parameter m for the C-1 and C-2 groups decreases first and then increases with strain rate, while the C-3 group shows a wavy fluctuation, and the C-4 group increases first and then decreases. This indicates that within the fracture porosity range of 0.3% to 0.45%, there exists a threshold beyond which the variations of *F*_0_ and *m* with loading rate are reversed.

Based on the data, when the strain rate increases from 0.001 s^−1^ to 0.05 s^−1^, the average value of *m* increases significantly from 4.23 to 10.23. Notably, at a strain rate of 0.05 s^−1^, m reaches an extreme value, indicating a pronounced increase of *m* under high strain rate conditions. At the same strain rate, there is a negative correlation between fracture porosity and *m*. The parameter *F*_0_ shows a fluctuating decreasing trend with increasing strain rate—for instance, its average value decreases from 28.04 at 0.001 s^−1^ to 26.87 at 0.05 s^−1^—although the overall trend is relatively smooth. Generally, higher fracture porosity corresponds to a lower *F*_0_.

In summary, high strain rates significantly enhance the m value, while increased fracture porosity suppresses *m*, indicating a competitive relationship between these two effects. Moreover, *F*_0_ is more evidently influenced by the coupled effects of strain rate and fracture porosity, and the variation patterns of *F*_0_ and *m* at a strain rate of 0.001 s^−1^ differ markedly from those at higher dynamic strain rates. Therefore, test samples at strain rates of 0.01 s^−1^, 0.03 s^−1^, and 0.05 s^−1^ can be selected for further analysis.

Assuming that the relationships between the parameters *F*_0_ and *m* with strain rate and fracture porosity can be described by a polynomial surface, they can be represented by the following function.

(16) $$z=\beta_{0}+\beta_{1}\varepsilon +\beta_{2}\eta +\beta_{3}\varepsilon^{2}+\beta_{4}\varepsilon \eta +\beta_{5}\eta^{2}+\beta_{6}\varepsilon^{2}\eta +\beta_{7}\varepsilon \eta^{2}+\beta_{8}\eta^{3},$$

where $\beta_{0},\beta_{1},\cdots \beta_{8}$ represents the coefficient and z denotes the parameter *F*_0_ or *m*.

Finally, the least-squares method is applied to optimize the model by minimizing the sum of squared residuals. Matlab (2023 b) is then used to input the original data points into the fitting model, producing the following visualization of the fitting performance. Given the nonlinear interdependence between fracture density and strain rate effects on parameters *F*_0_ and *m*, higher-order polynomial regression was deliberately constrained to mitigate overfitting risks. A third-order polynomial regression model was selected through systematic optimization, achieving adjusted R^2^ values of 0.970 and 0.896 for *F*_0_ and *m* respectively when correlating with fracture density-strain rate interactions. This modeling approach successfully balances predictive accuracy with parsimony while maintaining physical interpretability of the constitutive parameters.

**Figure 9:**
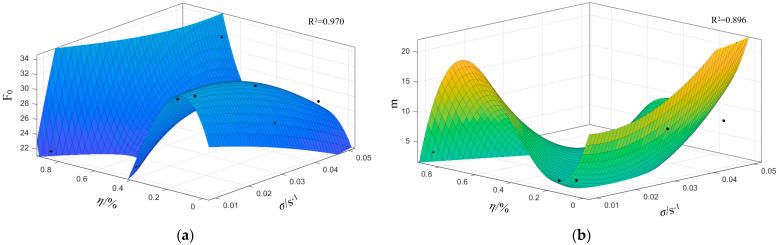
Fitting diagram of the parameters *F*_0_ and *m* polynomials: (**a**) *F*_0_; (**b**) *m*.

The coefficients for *F*_0_ and *m* and the revised computational results of the parameters are presented in Figure 9a and b respectively.

### 4.3. Model Verification

**Figure 10:**
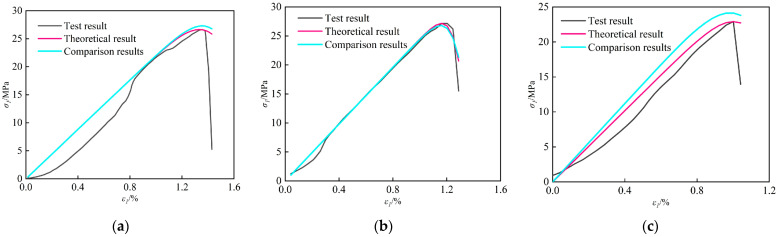

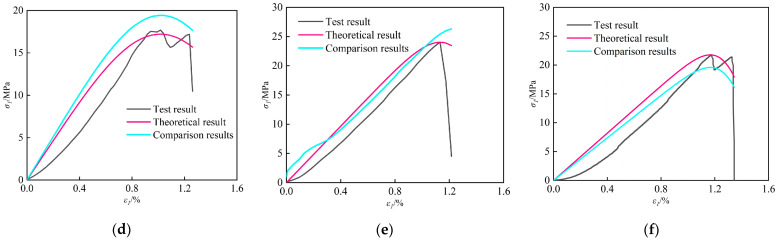
Coal sample tests and theoretical stress–strain curves: (**a**) C-1-3; (**b**) C-1-4; (**c**) C-2-4; (**d**) C-3-2; (**e**) C-3-4; (**f**) C-4-3.

By incorporating the modified parameters F_0_ and m from Table 5 into Equation (13), theoretical dynamic stress–strain curves of coal-rock were calculated for specimens subjected to strain rates of 0.01 s^−1^, 0.03 s^−1^, and 0.05 s^−1^. These results were compared with predictions from the reference model in literature [28] (which neglects fracture density effects) to evaluate model performance. Table 6 shows the key theoretical parameters of coal samples under dynamic load conditions. The correlation coefficients between experimental data and both models are summarized in Table 7, with comparative fitting results illustrated in Figure 10.

Based on the correlation between the Weibull distribution parameters *F*_0_ and *m* and the strain rate and fracture porosity, the corrected theoretical curves accurately describe the dynamic mechanical characteristics of the coal samples, such as uniaxial compressive strength and peak strain. This reveals the evolution of nonlinear failure behavior in coal under the coupled effects of strain rate and the pre-existing fracture network. By quantifying the feedback relationship between fracture structural defects and mechanical response during dynamic damage, the constructed strain rate–fracture porosity synergistic mechanism not only provides a reference for optimizing multi-factor coupled constitutive models but also holds significant engineering value for the prevention and control of dynamic hazards such as rock bursts in deep coal mining, thereby facilitating safe and efficient extraction.

Figure 10 demonstrates that specimen C-1-4 achieved optimal validation performance with R^2^ = 0.986, while C-2-4 exhibited the poorest agreement (R^2^ = 0.842). The mean R^2^ across all coal specimens reached 0.875. Table 5 and Table 6 reveals that specimen C-4-3 displayed maximum parameter deviations: 2.296% in compressive strength and 1.5% in elastic modulus. In contrast, specimens C-1-4, C-2-4, and C-3-4 showed minimal deviations, with computational errors for both mechanical parameters approximating zero. These results confirm the model’s high reliability in predicting compressive strength and elastic modulus.

The stress–strain curve morphologies exhibit significant dependence on both fracture density and strain rate. For specimens in Group C-1 with ultra-low fracture density, both analytical models demonstrate comparable predictive accuracy due to minimal fracture-induced mechanical heterogeneity. However, for the remaining three groups (C-2 to C-4) with elevated fracture densities, the fracture density- and strain rate-modified constitutive model achieves superior correlation coefficients (R^2^) with experimental measurements, demonstrating enhanced fidelity in capturing fracture-dominated nonlinear deformation responses.

## 5. Conclusions

1. The uniaxial compressive strength of coal-rock exhibits a positive correlation with increasing strain rate. The dynamic elastic modulus decreases by 11.9% in the strain rate range of 0.001–0.01 s^−1^ and increases with strain rate in the range of 0.01–0.05 s^−1^. Additionally, uniaxial compressive strength shows a negative correlation with fracture porosity, with the elastic modulus decreasing by 24.8% under extremely low strain rate conditions as fracture porosity increases; however, in the strain rate range of 0.01–0.05 s^−1^ the influence of fracture porosity is diminished. This suggests that, under the coupled effects of strain rate and fracture porosity, high strain rates can partially mitigate the degradation effect of fractures on stiffness, while compressive strength remains more sensitive to fracture porosity.
2. Within the strain rate range of 0.001 s^−1^ to 0.05 s^−1^, the fractal dimension of the fragmented coal-rock ranges from 1.0 to 1.3. As the strain rate increases, both the average mass of the ejected coal-rock fragments and the average fractal dimension of these fragments gradually rise, indicating a more heterogeneous fragment size distribution.
3. In the strain rate range of 0.001 s^−1^ to 0.01 s^−1^, there exists a threshold where the trends of the parameters *F*_0_ and *m* before and after 0.01 s^−1^ are reversed. Similarly, within the fracture porosity range of 0.3% to 0.45%, a threshold is observed such that under strain rates between 0.001 s^−1^ and 0.05 s^−1^, the variations of *F*_0_ and *m* with increasing strain rate are opposite for fracture porosities below 0.3% compared to those above 0.45%.
4. High strain rates markedly enhance the *m* value, whereas increasing fracture porosity suppresses *m*, indicating a competitive interaction between the two effects. In contrast, the parameter *F*_0_ is more prominently influenced by the combined effects of strain rate and fracture porosity.
5. Based on the relationship between the Weibull distribution parameters *F*_0_ and *m* and the strain rate, a polynomial linear regression model is employed to quantify the effects of strain rate and fracture porosity on these distribution parameters. This approach is used to revise the dynamic statistical damage constitutive model for coal-rock, and a method for determining the revised model parameters is proposed. Comparison with experimental results demonstrates that the theoretical curves produced by the model align well with the experimental data.

## Figures and Tables

**Table 1:** Test results of coal samples.

Serial Number	*ε*/s^−1^	*η*/%	*σ_max_*/MPa	*E*/GPa
C-1-1	0.001	0.0848	24.3	2.752
C-2-1		0.1396	23.8	2.551
C-3-1		0.3572	18.92	2.259
C-4-1		0.7737	17.06	2.123
C-1-2	0.01	0.0783	24.75	2.292
C-2-2		0.1749	22.39	2.123
C-3-2		0.4106	21.76	2.05
C-4-2		0.8785	15.03	2.068
C-1-3	0.03	0.007	26.65	2.206
C-2-3		0.1117	23.4	2.299
C-3-3		0.4427	21.24	2.328
C-4-3		0.5287	17.2	2.331
C-1-4	0.05	0.0581	27.18	2.252
C-2-4		0.1309	24.04	2.417
C-3-4		0.3241	22.92	2.547
C-4-4		0.6697	20.11	1.907

**Table 2:** ANOVA of crack ratio.

Fracture Group	Density Range	Mean	Variance
Ultra-Low	0~0.1%	0.05705	0.0012
Low	0.1%~0.2%	0.13928	0.0007
Medium	0.3%~0.45%	0.38365	0.0028
High	0.45%~0.9%	0.71265	0.0223

**Table 3:** Characteristics of projectile fragments in coal and rock mass.

Serial Number	*ε*/s^−1^	The Quality of the Crushed Coal in Each Block Range/mm				Projectile Block Mass/g	Average Fractal Dimension
		<5	5~16	16~25	25~31.5		
C-1-1	0.001	13.4	18.2	17.3	13	61.9	1.071
C-2-1		16.4	16.2	20.8	16	69.4	
C-3-1		15.1	17.6	15.3	14.5	62.5	
C-4-1		10	18.7	14.1	5.8	48.6	
C-1-2	0.01	15	27.2	16.6	16.8	75.6	1.077
C-2-2		10.9	22.3	20.6	17.5	71.3	
C-3-2		26.2	27.6	19.5	21	82.8	
C-4-2		16	20.2	18.4	14.2	68.8	
C-1-3	0.03	20.6	22.2	21.5	19.3	83.6	1.117
C-2-3		22.1	21.7	22.1	17.5	83.4	
C-3-3		16.7	27.4	23.8	23.5	91.4	
C-4-3		15.6	19.5	24.3	14.2	73.6	
C-1-4	0.05	31	29.3	27.1	25.6	113	1.265
C-2-4		22.2	28.8	22.1	21.2	94.3	
C-3-4		23.8	29.3	20.6	24.5	98.2	
C-4-4		20.3	26.3	21.2	26.5	94.3	

**Table 4:** Calculation results of the constitutive model.

Serial Number	*m*	*F*_0_
C-1-1	5.34	30.04
C-1-2	3.87	34.04
C-1-3	10.07	27.72
C-1-4	21.14	24.65
C-2-1	5.34	29.41
C-2-2	3.18	33.03
C-2-3	3.93	32.03
C-2-4	7.78	26.65
C-3-1	3.67	26.52
C-3-2	10.27	22.53
C-3-3	3.51	30.24
C-3-4	9.38	24.24
C-4-1	2.58	27.18
C-4-2	3.38	21.69
C-4-3	3.13	25.54
C-4-4	2.60	31.95

**Table 5:** The coefficients of *F*_0_ and *m* and the results of the calculations.

Serial Number	*m*	*F*_0_	Coefficient	*m*	*F*_0_
C-1-2	4.111	33.723	*β*_0_	4.111	33.723
C-1-3	10.959	27.856	*β*_1_	10.959	27.856
C-1-4	17.972	24.927	*β*_2_	17.972	24.927
C-2-2	2.653	33.513	*β*_3_	2.653	33.513
C-2-3	2.815	31.854	*β*_4_	2.815	31.854
C-2-4	12.502	26.180	*β*_5_	12.502	26.180
C-3-2	10.659	22.347	*β*_6_	10.659	22.347
C-3-3	2.310	26.650	*β*_7_	3.750	30.169
C-3-4	7.632	24.479	*β*_8_	7.632	24.479
C-4-2	3.283	21.713	/	/	/
C-4-3	3.352	25.571	/	/	/
C-4-4	2.799	31.897	/	/	/

**Table 6:** Key theoretical parameters of coal samples under dynamic load conditions.

Serial Number	Compressive Strength/MPa	Elastic Modulus/GPa	Compressive Strength Deviation/%	Elastic Modulus Deviation/%
C-1-3	27.290	2.200	2.40	0.27
C-1-4	27.176	2.252	0.01	0.00
C-2-4	24.040	2.417	0.00	0.00
C-3-2	21.746	2.046	0.06	0.20
C-3-4	22.918	2.547	0.01	0.00
C-4-3	17.662	2.296	2.69	1.50

**Table 7:** Correlation coefficients between model predictions and experimental data.

Serial Number	Modified Model	Reference Model
C-1-3	0.904	0.899
C-1-4	0.986	0.980
C-2-4	0.842	0.786
C-3-2	0.955	0.901
C-3-4	0.951	0.849
C-4-3	0.941	0.878

## Data Availability

The original contributions presented in this study are included in the article. Further inquiries can be directed to the corresponding author.
